# Effect of Combined Vitamin C and Thiamine Therapy on Myocardial and Inflammatory Markers in Cardiac Surgery: A Randomized Controlled Clinical Trial

**DOI:** 10.3390/nu17061006

**Published:** 2025-03-13

**Authors:** Mantana Saetang, Wirat Wasinwong, Maliwan Oofuvong, Jutarat Tanasansutthiporn, Laortip Rattanapittayaporn, Sutthasinee Petsakul, Pongsanae Duangpakdee, Puripong Rodneam, Parin Boonthum, Supphamongkhon Khunakanan, Chaitong Churuangsuk, Wilaiwan Sriwimol, Amphan Chantarokon, Kanjana Nuanjun, Dararat Yongsata

**Affiliations:** 1 Department of Anesthesiology, Faculty of Medicine, Prince of Songkla University, Songkhla 90110, Thailand; stmantana@gmail.com (M.S.); oomaliwa@gmail.com (M.O.); takiangka@hotmail.com (J.T.); laortip.r@gmail.com (L.R.); p.sutthasinee@gmail.com (S.P.); camphanster@gmail.com (A.C.); nkanjana@medicine.psu.ac.th (K.N.); kidinnocent_tt@hotmail.com (D.Y.); 2 Department of Surgery, Faculty of Medicine, Prince of Songkla University, Songkhla 90110, Thailand; pongsanae.d@psu.ac.th (P.D.); palm.phuri@gmail.com (P.R.); prinsx094@gmail.com (P.B.); bozo.sup@gmail.com (S.K.); 3 Department of Internal Medicine, Faculty of Medicine, Prince of Songkla University, Songkhla 90110, Thailand; chaitong.c@psu.ac.th; 4 Department of Pathology, Faculty of Medicine, Prince of Songkla University, Songkhla 90110, Thailand; sriwimol.wi@gmail.com

**Keywords:** cardiac biomarkers, cardiac surgery, myocardial injury, oxidative stress, thiamine, vitamin C

## Abstract

**Background:** Oxidative stress and systemic inflammation during cardiac surgery can lead to postoperative complications. Although vitamin C and thiamine (vitamin B1) have individually demonstrated protective effects, their combined effects remain underexplored. This study aimed to evaluate the efficacy of combined vitamin C and B1 therapy versus that of vitamin C alone in reducing inflammatory and cardiac biomarkers and improving postoperative outcomes in patients undergoing cardiac surgery. **Methods:** In this prospective, double-blind, randomized controlled trial, 64 patients scheduled for elective cardiac surgery at a tertiary care center were randomized to receive either 1000 mg vitamin C or a combination of 1000 mg vitamin C and 100 mg vitamin B1 at four perioperative time points. Primary outcomes included changes in inflammatory biomarkers [C-reactive protein, interleukin-6 (IL-6), and white blood cells], and cardiac biomarkers [creatine kinase-MB, Troponin-I, and lactate dehydrogenase]. Secondary outcomes included hemodynamic parameters and left ventricular function. **Results:** Compared with vitamin C alone, combined vitamin B1 and vitamin C significantly reduced postoperative cardiac biomarker levels. IL-6 levels were significantly lower immediately in the combined group; however, this effect was not sustained at 24 h post-surgery. Up to 24 h after surgery, no significant differences in hemodynamic stability or left ventricular ejection were observed between the groups. Notably, the combined therapy group demonstrated a lower incidence of postoperative arrhythmias and shorter dobutamine duration within 24 postoperatively. **Conclusions:** Combined vitamin C and B1 therapy significantly reduced markers of myocardial injury and early inflammatory responses (IL-6) in patients undergoing cardiac surgery, suggesting its potential as a protective agent.

## 1. Introduction

Cardiac surgery is vital in treating various cardiovascular diseases; however, it is often associated with significant complications, including low-cardiac-output syndrome (LCOS), infections, arrhythmias, and renal dysfunction [1,2,3,4]. These serious complications are associated with high healthcare costs and increased length of hospitalization [5].

Cardiopulmonary bypass (CPB) is a critical technology that allows temporary replacement of heart and lung functions during intricate surgical procedures. CPB may cause several biochemical changes in the microcirculation that can lead to a systemic inflammatory response [6,7]. Surgical incision, post-CPB reperfusion injury, and blood contact with non-endothelial membranes can activate inflammatory signaling pathways, producing and activating inflammatory cells with cytokine production and oxidative stress [7]. This inflammatory storm can cause damage to vital organs, especially the heart, leading to complications in the postoperative period [7,8,9].

Attenuating oxidative stress and the resulting inflammatory response may therefore represent a promising protective strategy, especially in high-risk cardiac surgery [10]. Vitamin C has been shown to exert protective effects by reducing reactive oxygen species (ROS) production, enhancing nitric oxide bioavailability, and modulating pro-inflammatory cytokine release. By scavenging free radicals, vitamin C helps limit oxidative damage to cardiomyocytes, thereby preventing apoptosis and endothelial dysfunction [11]. Additionally, it limits lipid peroxidation in the cell membrane and protects against myocardial damage caused by ischemic–reperfusion injury after surgery [11]. Furthermore, it can attenuate the pro-inflammatory response by inhibiting of nuclear factor-kappa light chain enhancer of activated B cells, therefore decreasing cytokine release [12]. Importantly, plasma vitamin C decreases by approximately 70% within 24 h after CPB and may remain low for up to 2 weeks [13]. Therefore, the serious reduction in vitamin C leads to the destruction of the body’s defense against activated oxygen species produced during cardiac surgery [13].

Similarly, thiamine (vitamin B1), as a scavenger of ROS, removes hydroxyl (HO^•^) radicals at significantly higher levels than hydroperoxyl radical (HOO^•^) [14]. Vitamin B1 enhances macrophage phagocytic activity, inhibits NF-κB activation, and protects neutrophil surface sulfhydryl groups from oxidative damage. Additionally, it regulates proinflammatory cytokine production in macrophages and interacts with the p53 suppressor protein, which controls cell proliferation, apoptosis, and death, indicating potential cytoprotective effects [15]. Beyond its antioxidant role, vitamin B1 plays a crucial role in improving tissue perfusion and stabilizing lactate levels, a key marker of ischemic–reperfusion injury [12,16]. In a thiamine-deficient state, impaired enzyme activity disrupts the Krebs cycle, leading to decreased adenosine triphosphate (ATP) synthesis, oxidative damage, and cell death [17]. A decrease in thiamine levels has been reported after cardiac surgery [18,19], which may contribute to cardiac dysfunction, increased oxidative stress, and endothelial damage [20]. Metabolic disturbances in thiamine deficiency lead to a metabolic acidosis, and laboratory evaluation will often reveal an elevated lactate concentration. Moreover, lower thiamine levels have been associated with increased mortality in critically ill patients [21].

Although previous studies have indicated individual benefits of vitamins C and B1 in mitigating oxidative stress and myocardial injury [22,23,24,25], existing studies are limited by heterogeneity and mostly focus on coronary bypass graft (CABG) surgery. Emerging evidence suggests that vitamin B1 and vitamin C may have synergistic effects in critically ill patients [26]. To address this gap, our study aimed to evaluate the combined effect of vitamins C and B1, compared with that of vitamin C alone, on postoperative outcomes in patients undergoing cardiac surgery. Specifically, we focused on the markers of myocardial injury such as creatine kinase-MB (CK-MB), Troponin-I, lactate dehydrogenase (LDH), and lactate.

## 2. Materials and Methods

### 2.1. Study Design

This was a prospective, double-blind, randomized controlled trial conducted at the Cardiothoracic Surgery Operating Theater, Songklanagarind Hospital, a tertiary care center in Southern Thailand.

### 2.2. Study Setting

This study was conducted in the cardiothoracic operating room and intensive care unit (ICU) of the Songklanagarind Hospital, Thailand. All the participants were enrolled from 15 October 2022 to 30 August 2024.

### 2.3. Study Population

#### 2.3.1. Inclusion Criteria

- Scheduled for elective, non-emergent open cardiac surgery;
- Aged 18 years or older;
- Left ventricular ejection fraction (LVEF) > 35%;
- No known coagulopathy before surgery.

#### 2.3.2. Exclusion Criteria

- Administration of vitamin C or thiamine within 7 days before surgery;
- Known allergies to vitamin C or thiamine;
- Indications for thiamine administration (such as chronic alcohol use);
- Participation in multiple simultaneous research studies;
- Autoimmune diseases or ongoing immunosuppressive therapy;
- History of renal calculi and risk of iron overload disorders (e.g., thalassemia and hemochromatosis);
- Preoperative creatinine clearance < 40 mL/min or serum creatinine > 1.8 mg/dL;
- Known bleeding disorders or current anticoagulant therapy;
- Active infection, malignancy, or tumor;
- History of atrial fibrillation;
- Pregnancy;
- Mechanical ventilation or vasopressor/inotropic use on the day of surgery.

### 2.4. Sample Size

The sample size was calculated based on the findings from a previous study [27]. The C-reactive protein (CRP) levels on the first postoperative day were reported to be 11 mg/dL in the combined vitamin C and B1 group and 18 mg/dL in the placebo group. The CRP level in the vitamin-C-only group was assumed to be approximately 15 mg/dL. Based on these assumptions, the sample size was calculated to be 29 participants per group. To account for a 10% dropout rate, the final required sample size was adjusted to 32 participants per group, resulting in a total of 64 participants.

### 2.5. Randomization and Blinding

The participants were randomized into two groups in a 1:1 ratio using a computer-generated randomization sequence. Allocation was concealed in sealed opaque envelopes and handled by research staff not involved in patient care. The medications were prepared in de-identified 50 mL solution bags to ensure indistinguishability and administered by blinded healthcare providers. Patient outcomes were also collected and assessed by staff who were unaware of the study.

### 2.6. Intervention and Control Groups

- Intervention group—vitamin C combined with vitamin B1 group (Group BC)—received 1000 mg vitamin C and 100 mg thiamine dissolved in 50 mL normal saline, administered intravenously over 30 min.
- Control group—vitamin C alone group (Group C)—received 1000 mg vitamin C dissolved in 50 mL normal saline with an equivalent fluid volume administered intravenously over 30 min.

Medications were administered at four time points: after anesthesia induction, after separation from CPB, 12 h postoperatively, and 24 h postoperatively.

### 2.7. Informed Consent and Ethical Approval

This prospective, randomized clinical controlled trial was approved by the Institutional Review Board of the Faculty of Medicine, Prince of Songkla University (REC.64-152-8-1; 12 October 2022). The Declaration of Helsinki was followed in the conduct of this study, and each participant provided written informed consent. This study was registered in the Thai clinical trial with the registration number TCTR20220430005 (https://www.thaiclinicaltrials.org/show/TCTR20220430005 accessed on 30 April 2022). This manuscript adheres to the applicable CONSORT guidelines.

### 2.8. Anesthesia and Surgical Protocols

Each patient was premedicated with 1000 mg of paracetamol orally for approximately 30–60 min before arriving at the operating theater. Anesthesia induction, intubation, and maintenance included the administration of midazolam, fentanyl, propofol, rocuronium, and sevoflurane. Pressure-controlled mechanical ventilation was set with 0.5 FiO_2_, 6–8 mL/kg tidal volume, 12–14 breaths/min respiratory rate, and 5 cm H_2_O positive end-expiratory pressure. All the patients received a bolus of 10 mg/kg tranexamic acid intravenously after intubation, followed by continuous infusion at a rate of 1 mg/kg/h until separation from the CPB. Intravenous heparin (3–4 mg/kg) was administered to maintain an activated clotting time of >400 s during CPB. CPB was performed using standard on-pump methods (mild hypothermia and blood cardioplegia), and protamine was administered for heparin reversal. Separation from CPB was achieved using vasopressors and/or inotropic agents based on the decision of the attending anesthesiologist and surgeon. Additionally, blood components (packed red blood cells, fresh frozen plasma, platelets, and cryoprecipitate) were transfused as appropriate to correct anemia and coagulopathy after weaning from CPB. Intravenous paracetamol (20 mg/kg) was administered 6 h after the premedicated oral dose. Tramadol (50 mg) was also administered intravenously after separation from the CPB, along with bupivacaine for local infiltration at the sternal incision. Subsequently, all the patients were transferred to the cardiac ICU postoperatively and were weaned from mechanical ventilation following the standard protocols. Intravenous patient-controlled analgesia (PCA) with morphine or fentanyl was administered to all the patients during the postoperative period. Postoperative complications manifesting within 24 h were also reviewed and recorded.

### 2.9. Intraoperative Monitoring

Intraoperative and postoperative hemodynamics were recorded at defined intervals, including systolic, diastolic, and mean arterial and central venous pressures. LVEF was assessed preoperatively, intraoperatively, and 24 h postoperatively using transthoracic and transesophageal echocardiography. Other standard monitoring methods included pulse oximetry, capnography, inspired gas concentration, electrocardiography, body temperature (via both the nasopharynx and rectum), arterial blood gas with electrolytes, activated clotting time, and urine output.

### 2.10. Outcomes

#### 2.10.1. Primary Outcome

To evaluate the effects on inflammatory biomarkers—CRP, interleukin-6 (IL-6), and white blood cells (WBC)—and cardiac biomarkers—CK-MB, Troponin-I, and LDH—we compared the group receiving combined vitamin C and B1 therapy with the group receiving vitamin C alone.

#### 2.10.2. Secondary Outcomes

- Hemodynamic parameters: To evaluate systolic blood pressure (SBP), diastolic blood pressure (DBP), and mean arterial pressure (MAP) from pre-induction through the 24 h postoperative period between the group with combined vitamin C and B1 and the group with vitamin C alone.
- Left ventricular function: To assess the percentage change in LVEF from the preoperative baseline to 24 h postoperatively, we compared the combined vitamin C and B1 group with the group with vitamin C alone.

### 2.11. Statistical Analysis

All statistical analyses were performed using R software (version 4.3.1, R Foundation for Statistical Computing, Vienna, Austria). Continuous variables were assessed for normality using the Shapiro–Wilk test. Normally distributed data are expressed as mean ± standard deviation (SD) and compared using an independent *t*-test. Non-normally distributed data are presented as median (interquartile range [IQR]) and were analyzed using the Mann–Whitney U test.

Categorical variables are expressed as frequencies (percentages) and analyzed using the chi-square test or Fisher’s exact test, as appropriate. For small sample sizes or when the expected frequencies were less than 5, Fisher’s exact test was applied.

For longitudinal outcomes (e.g., hemodynamic parameters and biomarker levels measured at multiple time points), comparisons between groups were performed using a repeated-measures analysis of variance (ANOVA) for normally distributed data or mixed-effects models to account for within-subject correlations over time. The models included fixed effects for group, time, and their interaction, as well as random intercepts for the participants.

Statistical significance was set at *p* < 0.05. All the tests were two-tailed. Missing data were handled using multiple imputations by applying a chained-equation approach to generate the imputed datasets. Sensitivity analyses were conducted to ensure the robustness of the results, and all key analyses were repeated on the complete case datasets for comparison.

## 3. Results

### 3.1. Baseline Characteristics

**Figure 1:**
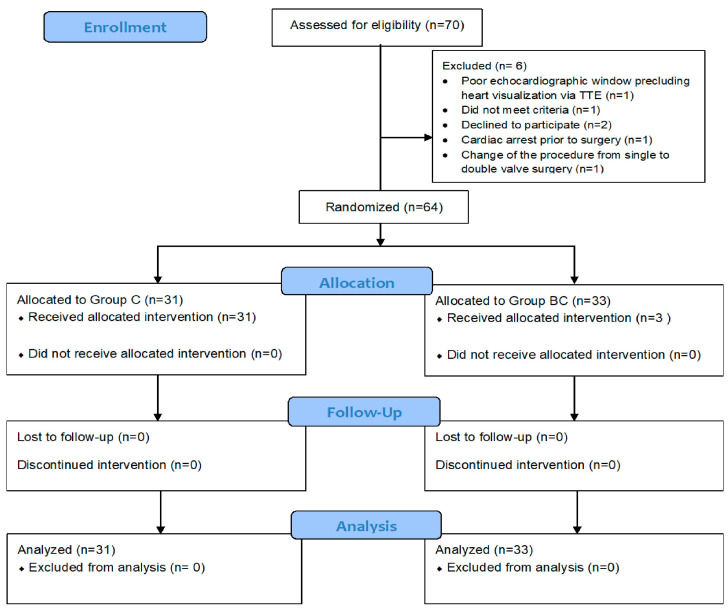
Consolidated Standards of Reporting Trials (CONSORT) diagram of the study. TTE, transthoracic echocardiogram; Group C, patients receiving vitamin C; Group BC, combined vitamin C and B1.

A total of 64 patients were randomized into two groups: Group C (*n* = 31) and Group BC (*n* = 33) (Figure 1). The two groups were comparable in terms of demographic data (Table 1), including median age (59 vs. 60 years, *p* = 0.814), sex distribution (male: 64.5 vs. 63.6%, *p* = 1.000), and body mass index (median 24.6 vs. 25.4 kg/m^2^, *p* = 0.727). The prevalence of comorbidities was similar between the groups. However, the use of angiotensin-converting enzyme inhibitors (44.8 vs. 16.1%, *p* = 0.032), diuretics (55.2 vs. 25.8%, *p* = 0.040), and statins (89.7 vs. 64.5%, *p* = 0.046) were significantly higher in Group C.

### 3.2. Inflammatory Biomarkers (Table 2)

**Figure 2:**
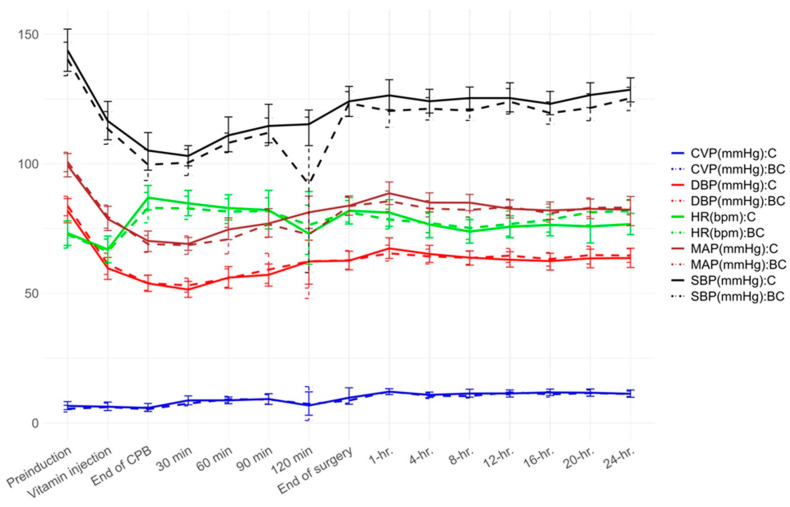
Hemodynamic parameters during intraoperative and postoperative periods in patients receiving vitamin C (Group C) and combined vitamin C and B1 (Group BC) while undergoing cardiac surgery. CVP, central venous pressure; DBP, diastolic blood pressure; HR, heart rate; MAP, mean arterial pressure; SBP, systolic blood pressure.

- CRP: Levels were similar between the groups at all time points, including 24 h postoperatively.
- IL-6: Group BC showed significantly lower levels immediately after surgery (median 63.1 vs. 114.4 pg/mL, *p* = 0.003), but no difference was observed at 24 h (*p* = 0.394) (Figure 2).
- WBC count was slightly lower in Group BC at 24 h postoperatively, but the difference was not statistically significant (mean 10,351.2 vs. 11,938.4 per microliter, *p* = 0.055).

**Table 2:** Perioperative cardiac biomarkers, inflammatory markers, and other laboratory results in patients receiving vitamin C (Group C) and combined vitamin C and B1 (Group BC) during cardiac surgery.

	Preoperative			Immediate Postoperative			24 h Postoperative		
Laboratory Results	Group C	Group BC	*p*-Value	Group C	Group BC	*p*-Value	Group C	Group BC	*p*-Value
CK-MB (IU/L), median (IQR)	1 (1, 1.2)	1 (1, 1.2)	0.928	22.1 (13.2, 27.6)	14.8 (9.3, 18.6)	0.008	12.8 (9.1,18.4)	7.9 (4.5, 15.6)	0.048
Troponin-I (ng/mL), median (IQR)	5.9 (3.8, 12.8)	4.4 (2.7, 12.3)	0.342	1606.8 (983.7, 2568.9)	618 (388.6, 1312.9)	0.005	3603.7 (2010.9, 6108)	1589.5 (683.5, 2865.9)	0.005
LDH (U/L), median (IQR)	184 (161, 202.5)	169 (151, 200)	0.39	332 (295.5, 361.5)	257 (230, 294)	<0.001	405 (335, 471)	330 (273, 384)	0.005
WBC (per microliter), median (IQR)	7360 (5960, 9310)	6580 (5970, 7750)	0.234	14,958.7 (6481.9)	13,256.7 (4841.4)	0.237	11,938.4 (3872.7)	10,351.2 (2520.4)	0.055
CRP (mg/L), median (IQR)	1 (1, 3.1)	1 (1, 2.2)	0.833	1.2 (1, 2.4)	1 (1, 2.1)	0.45	125.3 (36.7)	127.8 (47.6)	0.814
IL-6 (pg/mL), median (IQR)	3.7 (2.7, 6)	3.6 (1.8, 4.4)	0.245	114.4 (78.7, 177.4)	63.1 (47.2, 104)	0.003	99 (79.9, 125.6)	102.5 (85.1, 175.8)	0.394
Lactate (mmol/L), median (IQR)	1.1 (0.8, 1.4)	1 (0.9, 1.3)	0.989	2.8 (2, 3.9)	2.9 (2, 4)	0.078	2 (1.2, 2.5)	1.7 (1.4, 2)	0.431
Creatinine (mg/dL), mean (SD)	1 (0.2)	1 (0.3)	0.492	1 (0.2)	0.9 (0.2)	0.637	0.8 (0.7, 1.1)	0.9 (0.7, 1.1)	0.909
LVEF (%), mean (SD)	63.4 (10.9)	62.1 (11.3)	0.631	65.5 (13.3)	62.5 (11.9)	0.342	65.5 (11.2)	64.5 (9.8)	0.691

CK-MB, creatine kinase myocardial band; CRP, C-reactive protein; IL-6, interleukin-6; IQR, interquartile range; LDH, lactate dehydrogenase; LVEF, left ventricular ejection fraction; SD, standard deviation;; WBC, white blood cells.

### 3.3. Cardiac Biomarkers (Table 2)

- CK-MB: Group BC demonstrated significantly lower levels both immediately (median 14.8 vs. 22.1 IU/L, *p* = 0.008) and at 24 h postoperatively (median 7.9 vs. 12.8 IU/L, *p* = 0.048).
- Troponin-I: The levels were significantly reduced in Group BC immediately (median 618 vs. 1606.8 ng/mL, *p* = 0.005) and at 24 h after surgery (median 1589.5 vs. 3603.7 ng/mL, *p* = 0.005).
- LDH: Group BC showed significantly lower levels of LDH both immediately (median 257 vs. 332 U/L, *p* < 0.001) and 24 h after surgery (median 330 vs. 405 U/L, *p* = 0.005).

### 3.4. Lactate Levels (Table 2)

Lactate levels were similar between the groups preoperatively, immediately, and at 12 and 24 h postoperatively.

### 3.5. Hemodynamic Parameters (Figure 2)

No significant differences were observed in SBP, DBP, or MAP at any intraoperative or postoperative time point between the groups.

### 3.6. Left Ventricular Ejection Fraction (LVEF)

No significant differences were observed in LVEF between the two groups in the preoperative period, immediate postoperative state, or 24 h after surgery (Table 2). Both groups exhibited similar changes in LVEF from preoperative baseline to 24 h postoperatively (Group C: −2.1% vs. Group BC: −2.4%, *p* = 0.887).

### 3.7. Intraoperative Outcomes (Table 3)

- CPB time: Group BC had a significantly shorter mean CPB duration (107.8 vs. 128.6 min, *p* = 0.009).
- Arrhythmias: The incidence of arrhythmias during CPB was significantly lower in Group BC (0 vs. 16.1%, *p* = 0.022).

**Table 3:** Comparison of intraoperative variables between patients receiving vitamin C (Group C) and combined vitamin C and B1 (Group BC) during cardiac surgery.

Intraoperative Variables	Group C (*n =* 31)	Group BC *(n* = 33)	*p*-Value
Heart disease, *n* (%)			0.56
- Coronary artery disease	13 (41.9)	19 (57.6)	
- Mitral stenosis	2 (6.5)	1 (3)	
- Mitral regurgitation	8 (25.8)	7 (21.2)	
- Aortic stenosis	6 (19.4)	6 (18.2)	
- Aortic regurgitation	2 (6.5)	0 (0)	
Type of valvular surgery			0.854
- Valvular repair	6 (33.3)	6 (42.9)	
- Valvular replacement	12 (66.7)	8 (57.1)	
Cardiopulmonary bypass time (min), mean (SD)	128.6 (32.2)	107.8 (29.9)	0.009
Aortic cross clamp time (min), mean (SD)	87.1 (31)	74.3 (26.4)	0.08
Arrhythmia during CPB	5 (16.1)	0 (0)	0.022
Anti-arrhythmic agent requirement	1 (3.2)	0 (0)	0.484
Electrical defibrillation	3 (9.7)	0 (0)	0.108
Intraoperative inotropes, *n* (%)	31 (100)	32 (97)	1
- Epinephrine, *n* (%)	11 (35.5)	10 (31.2)	0.929
- maximum dose (mcg/kg/min), median (IQR)	0.2 (0.1, 0.2)	0.1 (0.1, 0.2)	0.178
duration (h), mean (SD)	1.4 (0.7)	1.5 (0.9)	0.73
- Norepinephrine, *n* (%)	17 (54.8)	18 (56.2)	1
- maximum dose (mcg/kg/min), median (IQR)	0.1 (0.1, 0.2)	0.1 (0.1, 0.2)	0.385
duration (h), mean (SD)	1.7 (1)	1.9 (1.2)	0.612
- Dobutamine, *n* (%)	24 (77.4)	23 (71.9)	0.829
- maximum dose (mcg/kg/min), median (IQR)	5 (5, 7.2)	5 (5, 6.5)	0.527
Duration (h), mean (SD)	1.5 (0.8)	1.3 (0.8)	0.211
Vasoactive-inotropic score			
- After separate CPB, median (IQR)	8 (5, 17.5)	10 (5, 15)	0.962
- At the end of surgery, median (IQR)	5 (0.8, 7)	5 (0, 6)	0.645
Intraoperative blood components			
- Packed red cell, *n* (%)	25 (80.6)	26 (78.8)	1
Packed red cell (units), median (IQR)	1 (1, 2)	1 (1, 2)	0.725
- Fresh frozen plasma, *n* (%)	30 (96.8)	32 (97)	1
Fresh frozen plasma (units), median (IQR)	3 (3, 3)	3 (3, 4.2)	0.526
- Platelet, *n* (%)	22 (71)	24 (72.7)	1
Platelet (units), median (IQR)	6 (6, 6)	6 (6, 6)	0.555

CPB, cardiopulmonary bypass; IQR, interquartile range; SD, standard deviation.

### 3.8. Postoperative Outcomes (Table 4)

The ICU and hospitalization, mechanical ventilation duration, and extubation time were not significantly different between the groups. However, the incidence of postoperative cardiac arrhythmias was significantly lower in Group BC compared with Group C (43.8 vs. 77.8%; *p* = 0.017). Moreover, the duration of postoperative dobutamine use was significantly shorter in Group BC compared with Group C (mean 7.7 ± 8.8 vs. 14.8 ± 8.5 h, *p* = 0.049).

**Table 4:** Postoperative clinical outcomes between patients receiving vitamin C (Group C) and combined vitamin C and B1 (Group BC).

Postoperative Clinical Outcomes	Group C (*n* = 31)	Group BC (*n* = 33)	*p*-Value
ICU stays (days), median (IQR)	3 (3, 4)	3 (3, 4)	0.701
Hospital stays (days), median (IQR)	8 (8, 10.5)	8 (7, 10)	0.407
Time to first extubation (h), median (IQR)	4.5 (1.1, 14.1)	4 (0, 13.8)	0.515
Duration of mechanical ventilation (h), median (IQR)	4.5 (1.1, 14.1)	4 (0, 13.8)	0.515
Non-invasive ventilation, *n* (%)	31 (100)	30 (90.9)	0.239
Postoperative complications, *n* (%)	27 (87.1)	32 (97)	0.19
Myocardial infarction	0	0	0.515
Cardiac arrest with ROSC	0	0	0.515
Cardiac arrhythmia	21 (77.8)	14 (43.8)	0.017
Neurological complications	2 (7.4)	2 (6.2)	1
Pulmonary complications	22 (81.5)	29 (90.6)	0.45
Renal complications	4 (14.8)	2 (6.2)	0.398
Postoperative inotropic agents	21 (67.7)	25 (75.8)	0.664
- Epinephrine, *n* (%)	5 (23.8)	6(24)	1
- maximum dose (mcg/kg/min), median (IQR)	0 (0, 0)	0.1 (0, 0.2)	0.714
duration (h), mean (SD)	2.6 (2.9)	7.1 (9.9)	0.35
- Norepinephrine, *n* (%)	15 (71.4)	15 (60)	0.617
maximum dose (mcg/kg/min), median (IQR)	0 (0, 0.1)	0 (0, 0.1)	0.676
Duration (h), mean (SD)	7.5 (7.5)	10.2 (8.6)	0.374
- Dobutamine, *n* (%)	14 (66.7)	12 (48)	0.33
- maximum dose (mcg/kg/min), mean (SD)	5.4 (2.4)	5.3 (3)	0.918
duration (h), mean (SD)	14.8 (8.5)	7.7 (8.8)	0.049
- Nitroglycerine, *n* (%)	4 (19)	3 (12)	0.686
maximum dose (mg/h), mean (SD)	7.5 (2.9)	10.3 (5.7)	0.421
duration (h), mean (SD)	13.8 (8)	12.8 (11.5)	0.9
Vasoactive-inotropic score, median (IQR)			
- At the end of surgery	1.4 (0, 5)	1.7 (0, 6)	0.592
- 6 h, median (IQR)	0 (0, 5)	0 (0, 3.3)	0.787
- 12 h, median (IQR)	0 (0, 3)	0 (0, 3.3)	0.891
- 24 h, median (IQR)	0 (0, 0)	0 (0, 0)	0.942
Blood transfusion within 24 h, *n* (%)	30 (96.8)	26 (78.8)	0.054

ICU, intensive care unit; IQR, interquartile range; SD, standard deviation; ROSC, return of spontaneous circulation.

## 4. Discussion

In this study, the addition of vitamin B1 to vitamin C significantly attenuated myocardial injury and immediate postoperative inflammatory responses, as evidenced by lower levels of CK-MB, Troponin-I, LDH, and IL-6 in patients undergoing cardiac surgery. However, the hemodynamic parameters, LVEF changes, and overall postoperative recovery times were not significantly different between the two groups. Combined therapy was associated with reduced postoperative arrhythmias and shorter duration of dobutamine use within 24 h after surgery.

The findings in this study align with literature demonstrating the benefits of vitamin C in reducing oxidative stress and inflammation and improving outcomes after cardiac surgery [28,29,30]. Vitamin C supplementation could reduce cardiac enzyme levels, inflammatory responses, arrhythmias, and ICU stay following CABG surgery [29,30,31,32]. However, thiamine addition represents a novel approach. Thiamine depletion is common in critically ill patients [21], and supplementation could improve outcomes in cardiac surgery.

Thiamine and vitamin C synergistically reduce organ injury in critical illnesses through distinct pathways. As a primary antioxidant, vitamin C scavenges free radicals, reduces reactive oxygen species production, and regenerates antioxidants. Thiamine, via thiamine pyrophosphate, generates nicotinamide adenine dinucleotide phosphate to support glutathione recovery and mitochondrial energy production. Together, they enhance cellular reducing power, mitigate mitochondrial dysfunction, protect the endothelial barrier, and reduce apoptosis, endothelial damage, and organ injury [12].

### 4.1. Cardiac Biomarkers

Cardiovascular surgeries—CABG and valve replacement—carry an inherent risk of myocardial injury due to ischemic–reperfusion injury and surgical trauma. Cardiac biomarkers are sensitive indicators of myocardial injury, with elevated levels being associated with worse surgical outcomes, including LCOS, prolonged ventilation, and mortality [33,34].

No significant changes were found in cardiac biomarkers, including CK-MB, Troponin I, and LDH over time between the high-dose vitamin C and normal saline groups [25]. However, our findings suggest that combined therapy may mitigate ischemic–reperfusion injury, a major cause of myocardial damage during cardiac surgery involving CPB.

### 4.2. Inflammatory Response

Assessment of the magnitude of the systemic inflammatory response may be considered complex because all tissues and organs display changes. However, acute-phase proteins have been considered ideal, since they are produced only by the liver in response to pro-inflammatory cytokine production at tissue injury sites, especially IL-6 [35]. Of these, CRP is particularly useful as it is sensitive to tissue injury, well standardized, routinely clinically measured worldwide, and reflects the magnitude of surgical injury [36]. High levels of perioperative inflammatory biomarkers were associated with increased postoperative mortality, stroke, nonfatal myocardial infarction, congestive heart failure, and major adverse cardiovascular events [37,38]. Tracking inflammatory biomarkers in cardiac surgery could provide valuable insights into a patient’s inflammatory status and potential for postoperative complications.

Vitamin C (2 g ascorbic acid IV) after anesthesia induction did not reduce IL-6 or CRP levels in patients undergoing CABG [32]; similar results were observed in our study. Notably, IL-6 levels were significantly lower in the combined therapy group immediately after surgery, suggesting attenuation of the early inflammatory response. However, 24 h postoperatively, this difference was no longer statistically significant (*p* = 0.394).

### 4.3. Lactate

Lactate elevation—a marker of anaerobic metabolism—is commonly observed after major cardiac surgery, and elevated postoperative lactate levels were associated with increased morbidity and mortality [39,40]. Elevated lactate levels are typically associated with impaired oxygen delivery, increased anaerobic metabolism, and ischemic–reperfusion injury [41], all of which are common during the perioperative period of cardiac surgery.

Andersen et al. [23] found no differences in postoperative lactate levels or clinical outcomes between patients receiving thiamine and placebo. Lomivorotov et al. [42] also reported that high-dose intravenous thiamine did not result in significant differences in key biological or physiological markers, including postoperative lactate levels, creatinine, cardiac index, or vasopressor/inotropic requirements, compared with placebo. Similarly, our study showed no significant differences in lactate levels between the combined therapy and vitamin C groups at any of the measured time points.

### 4.4. Postoperative Outcomes

The observed reduction in the dobutamine use duration in our study aligns with the findings of previous studies demonstrating the benefits of combined vitamin therapy in cardiac surgical patients. Balakrishnan et al. [43] reported that a combination of vitamin C, thiamine, and hydrocortisone significantly reduced vasopressor requirements in adult cardiac surgical patients with septic shock, highlighting the potential of this therapeutic strategy for enhancing cardiac stability and hemodynamic performance. Similarly, Athanasiou et al. [44] found that vitamin C supplementation reduced inotropic demand in cardiac surgery, further supporting the role of antioxidant therapy in optimizing postoperative management. Contrastingly, Lomivorotov et al. [42] reported that high-dose intravenous thiamine alone did not lead to significant differences in vasopressor or inotropic requirements compared with placebo. This discrepancy underscores the importance of the synergistic effects of vitamin C and thiamine, suggesting that a combination, rather than thiamine alone, may be necessary to achieve measurable clinical benefits.

Emadi et al. [25] found that vitamin C significantly improved LVEF at 72 h after surgery. Additionally, vitamin C could reduce the length of hospitalization [25,30]. However, in our study, no significant differences were observed in hemodynamic stability, LVEF changes, or length of ICU stay between the groups.

A meta-analysis demonstrated that vitamin C administration is effective as a prophylaxis for the prevention of postoperative atrial fibrillation (POAF) [31,45]. Shi et al. [46] reported that vitamin C alone or as an adjunct to other therapies can prevent POAF in patients undergoing cardiac surgery. Contrastingly, our study showed that combination therapy alone had a significantly lower incidence of intraoperative and postoperative arrhythmias. Eskandari et al. [32] showed that ascorbic acid (2 g vitamin C IV after anesthesia induction) did not reduce post-CABG atrial fibrillation.

### 4.5. Limitations

The sample size may limit the generalizability of findings and reduce the statistical power to detect subtle differences in secondary outcomes. Additionally, the study focused exclusively on short-term outcomes, with no long-term follow-up to evaluate the sustained effects of intervention. Variability in baseline medication use between groups may have introduced confounding factors, potentially influencing the results. Furthermore, fixed intervals for biomarker measurements may not fully capture dynamic changes, particularly during critical perioperative phases, thereby limiting the granularity of data. Another limitation is the absence of a vitamin B1-only group, making it difficult to determine whether the observed effects were due to vitamin B1 alone or the combined therapy. Future studies should consider a three-arm design (vitamin C alone, vitamin B1 alone, and their combination) to better understand the independent contribution of each vitamin.

## 5. Conclusions

The addition of vitamin B1 to vitamin C in patients undergoing cardiac surgery significantly reduced the markers of myocardial injury (CK-MB, Troponin-I, and LDH) and immediate postoperative inflammatory response (IL-6), underscoring its potential as a complementary therapeutic strategy. These findings highlighted the benefits of mitigating oxidative stress and inflammation during the perioperative period. Although no significant differences were observed in hemodynamic stability, changes in LVEF, or length of ICU stay, combination therapy was associated with a reduction in postoperative arrhythmias and a shorter duration of dobutamine use. Further research is warranted to explore the long-term effects, optimal dosing regimens, and impact of this combined therapy on critical outcomes such as mortality and long-term cardiac function.

## Figures and Tables

**Table 1:** Characteristics of the patients receiving vitamin C (Group C) and combined vitamin C and B1 (Group BC) during cardiac surgery.

Patient Characteristics	Group C (*n* = 31)	Group BC (*n =* 33)	*p*-Value
Age (years), median (IQR)	59 (53, 65.5)	60 (54, 65)	0.814
Sex			1
Male, *n* (%)	20 (64.5)	21 (63.6)	
Female, *n* (%)	11 (35.5)	12 (36.4)	
BW (kg), mean (SD)	64.4 (13)	66.6 (12.1)	0.483
BMI (kg/m^2^), median (IQR)	24.6 (22.8, 26.6)	25.4 (22.9, 27.2)	0.727
Comorbidity, *n* (%)	23 (74.2)	27 (81.8)	0.664
Ischemic heart disease	7 (30.4)	9 (33.3)	1
Heart failure	0 (0)	1 (3.7)	1
Hypertension	16 (69.6)	14 (51.9)	0.325
Dyslipidemia	18 (78.3)	17 (63)	0.386
Diabetes	10 (43.5)	12 (44.4)	1
Insulin use	0 (0)	1 (3.7)	1
Chronic lung disease	2 (8.7)	1 (3.7)	0.588
CVD (previous stroke or TIA)	2 (8.7)	0 (0)	0.207
Smoking	4 (17.4)	5 (18.5)	1
Current medications, *n* (%)	29 (93.5)	31 (93.9)	1
Beta-blocker	18 (62.1)	20 (64.5)	1
Calcium channel blocker	9 (31)	11 (35.5)	0.927
ACEI	13 (44.8)	5 (16.1)	0.032
ARB	5 (17.2)	5 (16.1)	1
Diuretics	16 (55.2)	8 (25.8)	0.04
Digoxin	1 (3.4)	0 (0)	0.483
Nitrate	11 (37.9)	15 (48.4)	0.578
Anticoagulant	1 (3.4)	0 (0)	0.483
Aspirin	18 (62.1)	22 (71)	0.648
Statin	26 (89.7)	20 (64.5)	0.046
ASA-PS class, median (IQR)	3 (3, 3)	3 (3, 3)	0.533
EuroScore-II (%), median (IQR)	0.9 (0.8, 1.3)	0.9 (0.8, 1.2)	0.444
NYHA class, median (IQR)	2 (2, 2)	2 (2, 2)	0.844
LVH by ECG, *n* (%)	22 (71)	13 (39.4)	0.022

ACEI, angiotensin-converting enzyme inhibitor; ARB, angiotensin receptor blocker; ASA-PS, American Society of Anesthesiologist–Physical Status classification; BMI, body mass index; BW, body weight; CVD, cerebrovascular disease; ECG, electrocardiogram; IQR, interquartile range; LVH, left ventricular hypertrophy; NYHA, New York Heart Association; SD, standard deviation; TIA, transient ischemic attack.

## Data Availability

The original contributions presented in this study are included in the article. Further inquiries can be directed to the corresponding author.

## Abbreviations

The following abbreviations are used in this manuscript:

LCOS	Low cardiac output syndrome
CPB	Cardiopulmonary bypass
CABG	Coronary bypass graft
CK-MB	Creatine kinase-MB
LDH	Lactate dehydrogenase
ICU	Intensive care unit
IL-6	Interleukin -6
PCA	Patient controlled analgesia
CRP	C-reactive protein
WBC	White blood cells
HR	Heart rate
SBP	Systolic blood pressure
DBP	Diastolic blood pressure
MAP	Mean arterial pressure
LVEF	Left ventricular ejection fraction
TTE	Transthoracic echocardiogram
ACEI	Angiotensin-converting enzyme inhibitor
ARB	Angiotensin receptor blocker
ASA-PS	American Society of Anesthesiologist-Physical Status classification
BMI	Body mass index
BW	Body weight
CVD	Cerebrovascular disease
CVP	Central venous pressure
ECG	Electrocardiogram
IQR	Interquartile range
LVH	Left ventricular hypertrophy
NYHA	New York Heart Association
SD	Standard deviation
TIA	Transient ischemic attack
ROSC	Return of spontaneous circulation
POAF	Postoperative atrial fibrillation
